# “Usability of data integration and visualization software for multidisciplinary pediatric intensive care: a human factors approach to assessing technology”

**DOI:** 10.1186/s12911-017-0520-7

**Published:** 2017-08-14

**Authors:** Ying Ling Lin, Anne-Marie Guerguerian, Jessica Tomasi, Peter Laussen, Patricia Trbovich

**Affiliations:** 1 Institute of Biomaterials and Biomedical Engineering, University of Toronto, Rosebrugh Building (RS), 164 College Street, Room 407, Toronto, ON M5S 3G9 Canada; 2 Department of Critical Care Medicine, The Hospital for Sick Children, Canada, 555 University Ave., 2nd Floor, Atrium - Room 2830A, Toronto, ON M5G 1X8 Canada; 3 Neurosciences and Mental Health Research, The Hospital for Sick Children Research Institute, Peter Gilgan Centre for Research & Learning, 686 Bay Street, Toronto, ON M5G 0A4 Canada; 4 Institute of Health Policy, Management and Evaluation, University of Toronto, 155 College St., Suite 425, Toronto, ON M5T 3M6 Canada; 5 Research and Innovation, North York General Hospital, 4001 Leslie Street, Toronto, ON M2K 1E1 Canada

## Abstract

**Background:**

Intensive care clinicians use several sources of data in order to inform decision-making. We set out to evaluate a new interactive data integration platform called T3™ made available for pediatric intensive care. Three primary functions are supported: tracking of physiologic signals, displaying trajectory, and triggering decisions, by highlighting data or estimating risk of patient instability. We designed a human factors study to identify interface usability issues, to measure ease of use, and to describe interface features that may enable or hinder clinical tasks.

**Methods:**

Twenty-two participants, consisting of bedside intensive care physicians, nurses, and respiratory therapists, tested the T3™ interface in a simulation laboratory setting. Twenty tasks were performed with a true-to-setting, fully functional, prototype, populated with physiological and therapeutic intervention patient data. Primary data visualization was time series and secondary visualizations were: 1) shading out-of-target values, 2) mini-trends with exaggerated maxima and minima (sparklines), and 3) bar graph of a 16-parameter indicator. Task completion was video recorded and assessed using a use error rating scale. Usability issues were classified in the context of task and type of clinician. A severity rating scale was used to rate potential clinical impact of usability issues.

**Results:**

Time series supported tracking a single parameter but partially supported determining patient trajectory using multiple parameters. Visual pattern overload was observed with multiple parameter data streams. Automated data processing using shading and sparklines was often ignored but the 16-parameter data reduction algorithm, displayed as a persistent bar graph, was visually intuitive. However, by selecting or automatically processing data, triggering aids distorted the raw data that clinicians use regularly. Consequently, clinicians could not rely on new data representations because they did not know how they were established or derived.

**Conclusions:**

Usability issues, observed through contextual use, provided directions for tangible design improvements of data integration software that may lessen use errors and promote safe use. Data-driven decision making can benefit from iterative interface redesign involving clinician-users in simulated environments. This study is a first step in understanding how software can support clinicians’ decision making with integrated continuous monitoring data. Importantly, testing of similar platforms by all the different disciplines who may become clinician users is a fundamental step necessary to understand the impact on clinical outcomes of decision aids.

**Electronic supplementary material:**

The online version of this article (doi:10.1186/s12911-017-0520-7) contains supplementary material, which is available to authorized users.

## Background

The Intensive Care Unit (ICU) setting is a complex socio-technical environment where patients with life-threatening conditions, frequently needing advanced organ support technologies, are continuously monitored by teams of specialized clinicians [1, 2]. This setting is synonymous with multimodal monitoring (MMM) defined as “the combined use of monitors, including […] clinical examination, laboratory analysis, imaging studies, and physiological parameters” and relies on human knowledge and skills to effectively use the data [3–6]. However, the massive amount of data may not be serving patient outcomes. Clifford reports a “growing awareness within medical communities that the enormous quantity and variety of data available cannot be effectively assimilated and processed without automated or semi-automated assistance” [7]. Celi attributes the difficulty of establishing cause and effect relationships between the interventions and the critically-ill patients to the “exceptional complexity of the [ICU] environment […] particularly vulnerable to variation across patient subsets and clinical contexts” [8]. In pediatric intensive care, complexity of care is increased compared adults due to weight-based dosing, and age-dependent pharmacokinetics, pharmacodynamics, and physiological norms [9, 10]. Multidisciplinary team care that complements physician care has improved survival of this complex patient population [11]. In fact, the Society of Critical Care Medicine maintains that “Right Care, Right Now™” is best provided by an integrated team of dedicated medical experts [12]. In the Canadian setting, the core team is comprised of physician intensivists, nurses, and respiratory therapists. Consequently, all these clinicians must be able to effectively detect and react to changes in patient status informed by the vast array of MMM data. As such, we propose that data integration and visualization software may be a solution to help clinicians process MMM data. The study’s purpose was to test the data integration and visualization software, specifically the level of simplicity to detect and understand changes in the patient state. We hypothesize that to properly display patient-specific ICU data in a manner which conveys meaning to the clinician, software should support data processing in a thoughtful, intuitive, and user-friendly manner [13]. Sub-optimal care may be traced to “flawed user interfaces” that result in cognitive errors and data misinterpretation [14–16]. A human factors study approach was chosen to empirically identify ease of use and safety issues. This approach is well established in aviation and nuclear power industries to help inform what an optimal user-interface design is and has recently been applied to healthcare [17–20]. In this study, we tested the usability of T3™, a data integration and visualization software program. The study is the first to report the usability of a commercially available, interactive, data integration, and visualization software for an ICU setting.

### Data integration and visualization software

**Fig. 1:**
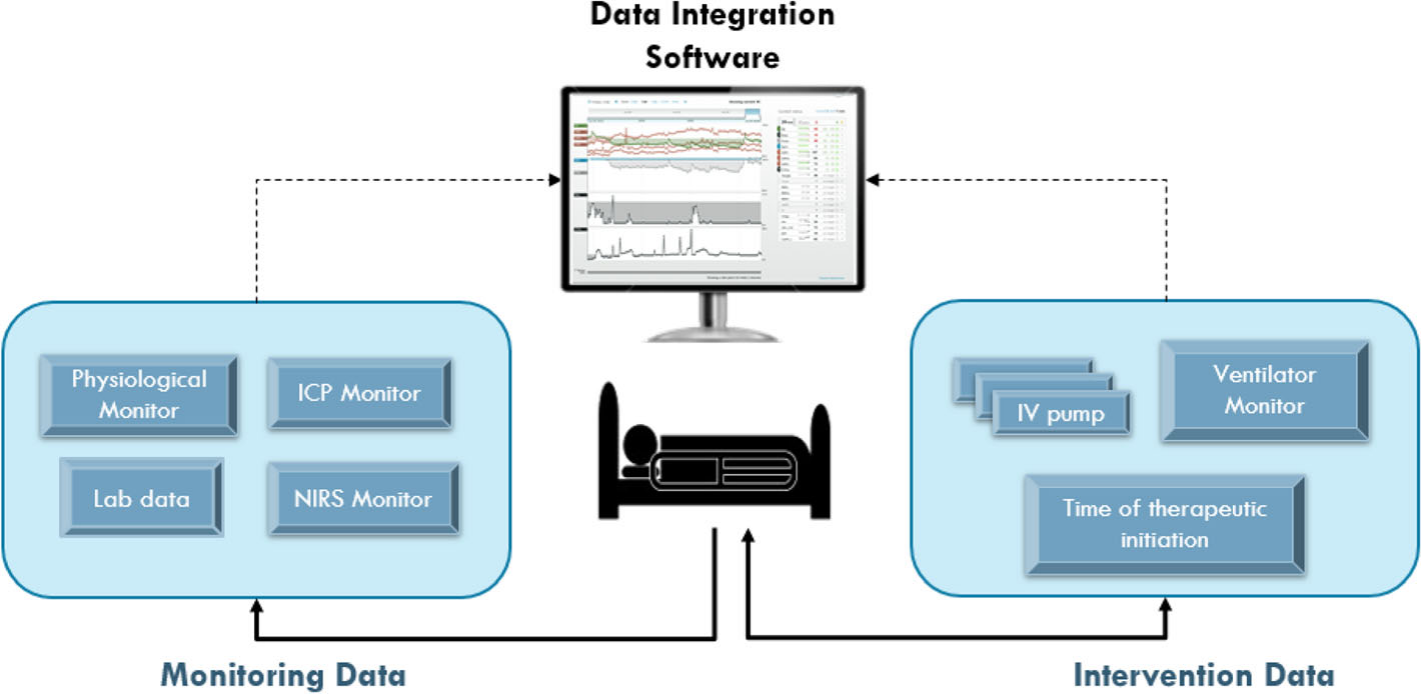
Examples of integrated monitoring and medical information data into a single software platform. Schematic of patient data sources available to the clinician participant during usability testing simulations. Monitoring data, generated from the patient, were detected through several monitoring medical devices, with sources shown on the left-side of the schematic. Intervention data, generated from therapeutic medical devices, are shown on the right-side of the schematic. Both types of data were available as continuous data streams, spanning days or weeks of the patient stay in the intensive care unit (ICU), and numbered in the dozens. ICP: intracranial pressure, NIRS: near-infrared spectroscopy, and IV: intravenous

In March of 2013, the T3™ software was implemented in a large pediatric ICU department. This web-based tool captures and displays integrated physiologic data exported from devices and monitors attached to patients. Specifically, it displays patient-generated physiological data and therapeutic intervention data (e.g. from infusion pumps and/or a ventilator, or diagnostic results from blood work with timestamps of important medical events such as chest closures or cardiac arrests). A schematic of data sources is presented in Fig. 1. Its three main functions are tracking (e.g. supports tracking of patient parameters to their unique norms over time), trajectory (e.g. visually integrates patient-specific data to show relationships), and triggering (e.g. derives meaning to support clinical decision-making through real-time computation of the data). It was available to all clinicians in the unit to either use in real-time or at a later point for review and debriefing.

To access T3™, a login separate from the existing hospital-based network is required. The interface is not permanently displayed, requiring the clinician to login and access the integrated data. Prior to implementation, the clinicians were shown the T3™ platform and were provided information about access and function. However, expectations for use within the ICU workflow were not made. It should also be noted that T3™ is not an approved patient monitor and there are no alarms incorporated into the software. It is used at the discretion of clinicians rather than mandated.

### Overview of project phases

**Fig. 2:**
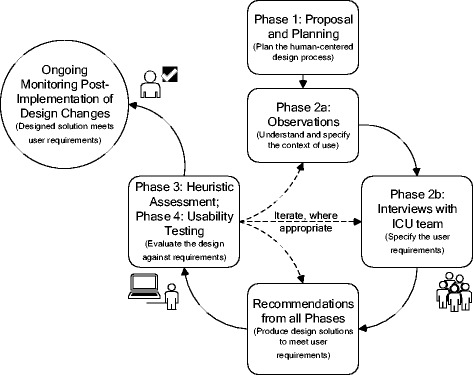
User-centered design and evaluation process of an existing data integration and visualization platform in accordance with the ISO 9241–210 standard. The iterative design and evaluation cycle is broken down into phases with the related ISO 9241–210 standard’s phases in parenthesis. The cycle was carried out once with each phase described for the design/evaluation of data integration and visualization software for intensive care monitoring and decision-making. Phase 1 was an initial phase where the user-centered design process was identified and work included gathering existing studies in the form of a systematic review. Phase 2 included both unit-level observations and clinician-level interviews to gather information about intensive care work using continuous data. Phase 3 was a heuristic assessment of the software to determine usability issues that violate accepted interface design principles and to suggest design solutions. Phase 4 was a usability test method where issues were identified by actual users performing true-to-work tasks and recommendations for design solutions were provided. Results from this last phase are presented here

To evaluate the T3™ continuous multimodal monitoring software design, specifically regarding end-user needs, a four-phase project was undertaken. The four phases included a systematic literature review, a qualitative study of the ICU and its clinicians, a heuristic evaluation of the software, and, finally, this usability investigation of the software (see full description in Fig. 2). All phases were part of the user-centered design and evaluation process. The systematic review focused on studies evaluating intensive care data integration and visualization on the clinician end-user. This review identified and assessed human factors studies of qualitative and quantitative natures. The second phase was an observational study in the ICU where clinicians were observed and interviewed to assess how physicians, nurses, and respiratory therapists used data, information, and technologies to influence critical decisions. The third phase of the project was a heuristic evaluation, which is a cost-effective usability technique. It identifies potential usability issues and associates them to violations of established good interface design principles [21]. Two human factors specialists, a senior ICU nurse and a senior ICU physician, found 50 potential usability issues associated with 194 heuristic violations [22]. While heuristic evaluation is an efficient and inexpensive method to uncover potential usability issues, usability testing is recognized as a better method because obstacles are obtained directly from the end-user’s interaction with the system. The fourth phase was a usability study, of which results are presented here. The goal of this final phase is to assess how existing data integration software can support physicians, nurses, and respiratory therapists with their use of continuous data.

Usability testing has been used to evaluate a number of healthcare technologies such as infusion pumps, computerized physician order entry systems, radiation therapy systems, and electronic medical record systems [10, 23–27]. There has been little focus on usability testing of data integration software from MMM devices [28, 29]. By observing users as they carry out realistic tasks, human factors specialists evaluate how technology helps users accomplish their work goals while assessing their needs and satisfaction. The strength of usability testing stems from the qualitative information revealed while using the software. Through these observations, human factors specialists identified the following: 1) what content is missing, and 2) what design elements went undetected, led to confusion, and/or led to errors. Based on this data, the design can be refined to provide better support mechanisms. Consequently, corrective actions are primarily system-based as opposed to changing human behavior.

The objectives of this study were to identify and evaluate usability issues of the data integration software and to determine the ease of use and potential safety impact on clinical decision-making, while considering the different perspectives of the multidisciplinary critical care team. In addition, recommendations to improve this and similar data integration platforms are provided.

## Methods

### Study design

This is a human factors usability study to assess specific continuous monitoring data integration software. The study was approved by the Research Ethics Board of the test site institution and the clinician participants’ hospital.

### Setting

Testing sessions were conducted from January to February of 2016 in a usability laboratory equipped with observational booths behind one-way glass and multiple ceiling-mounted cameras and microphones. During the two-month study period, internal data showed low usage with an average of 10 weekly users. Physicians used the software most of the time (96%) compared to nurses (4%) and respiratory therapists (0%). Usage logs from the ICU indicated there were between five and 17 weekly users, or approximately 6% of an over 300-clinician staff. The active users were mostly physicians, and they collectively used the software a total of 30 h per week.

### Software

**Fig. 3:**
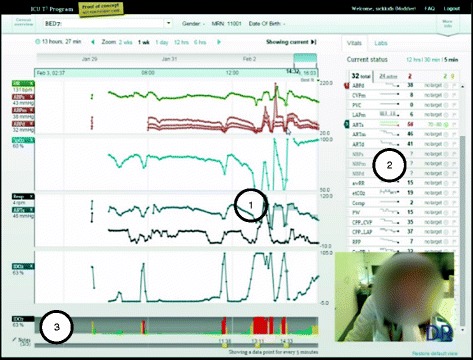
Representation of time series fictitious data and triggering visual aids: 1) shading, 2) sparklines, and 3) bar graph of single indicator IDO_2_ algorithm. Composite screenshot showing time series (*center*, all parametric trends), the primary visual aid, with 1) overlaid out-of-range target shading (*third graph area*), 2) sparklines showing condensed trend line of fixed time period with exaggerated minima and maxima (*far-right*), and 3) bar graphs representing the single indicator which calculates the risk of inadequate oxygen delivery (IDO_2_) (*bottom*)

T3™ is a web-based software available at multiple tertiary hospitals in North America, which continuously collects, integrates, and displays data from monitoring and intervention devices every five seconds. Four types of visual aids are generated: 1) time series of continuous numerical data (e.g. trend lines) displayed as an average over five seconds, 2) colored highlighted layer over time series (e.g. shading of trend lines), 3) automatic short-term trends (e.g. sparklines), and 4) persistent bar graph representation of percent risk (e.g. IDO_2_ indicator). All are shown in Fig. 3. We tested T3™ version 1.6 as a fully-interactive working prototype software, identical to what was available in the ICU. The version we tested included a 16-parameter proprietary algorithm which estimated the risk of inadequate oxygen delivery [30]. Software was accessed through an intranet website, hosted on a virtual server behind the hospital’s firewall, and used a Google Chrome™ web browser installed on a computer running a Microsoft® Windows™ operating system. TechSmith® Morae® software version 2.0.1 was used to collect audio and video data from the computer screen and the participant’s facial expressions as they interacted with the software during the simulations (See Fig. 3). R software version ×64 3.2.2, package irr, function kappa2, was used to calculate statistics.

### Scenarios and tasks

Scenarios, of which there were three, were based on post-cardiac surgery newborn patients, their data sets, and the events they experienced while in the unnamed North American pediatric hospital’s ICU. The comprehensive data sets included dozens of monitoring and intervention data streams and were good representations of closely monitored ICU patients. The data sets, provided by the software developers, were populated with fictitious names, medical record numbers, and background information (See Table 1). During each test session, the data replayed from the same start time and presented the patient’s evolving status in real-time. Each scenario contained at least 24 parameters of continuously collected data, and clinicians could simultaneously visualize data from up to 16 parameters (four per panel).

**Table 1:** Description, parameters available, key data features of three scenarios, based on real patients, used to test the T3™ software functions

Scenario number	Main events or interventions	Number of parameters	Key data features
1	- 2 episodes of hypotension - 1 cardiac arrest - 1 initiation onto extracorporeal membrane oxygenation	32 total 24 active	- physiological monitoring - infusion pump data - temperature data - laboratory data
2	- 1 increased erroneous, medical infusion (dopamine) - 1 intervention (inhaled nitric oxide therapy)	34 total 28 active	- physiological monitoring - infusion pump data - laboratory data
3	- 1 attempt at bedside chest closure - 1 cardiac arrest	46 total 36 active	- physiological monitoring - infusion pump data - ventilator data - three oxygen saturation parameters - laboratory data

These three scenarios were the overall context in which participants were asked to carry out 20 types of tasks regarding continuous data use. The tasks are described in Additional file 1.

### Participants

Participants were pediatric intensive care clinicians from three critical care disciplines: seven physicians, eight nurses, and seven respiratory therapists. They were from the same institute where the software was implemented. They were the equivalent of full time staff of a large, tertiary, Canadian, pediatric hospital and all had access to T3™ in their ICU. To detect at least 80% of possible discipline-specific usability issues, seven participants from each discipline were sufficient [31].

### Procedure

Upon arriving to the simulation lab, each participant received a brief orientation, outlining the purpose and objectives of the evaluation, and consent was formally obtained. Participants were informed that they would be observed, videotaped, and audiotaped. The study facilitator addressed any questions or concerns before the participants reviewed and signed the consent form. Participants then completed the pre-test questionnaire. No training was provided before the experiment, although some clinicians received introductory training sessions post ICU software launch.

During the simulations, participants were asked to “think aloud” as they executed each task. This was to gain insight into their thought process, as well as providing insight into their use of data and the information available to them. Both audio and video recordings were made of simulations (See Fig. 3). When a participant was challenged, they verbalized their thoughts to indicate the cause. A facilitator and two data recorders were in the observation room behind a one-way mirror. They facilitated, observed, and recorded participant performance (e.g. use, difficulties, and errors) and feedback. After participants completed scenarios or the allotted time was exhausted, the facilitator conducted a debrief interview and a post-test questionnaire. Feedback about participant experience with the T3™ system was collected, comments during simulation were clarified, and any concerns and/or questions arising from the evaluation were addressed.

### Data analysis

#### Scoring task completion and usability error definition: Use error rating

Within this study, we established Use Error Ratings (UERs) on a scale of 2–0 to assess clinicians’ software competency. (UER definitions are presented in Table 2, both in nominal form and as numerical codes). Two means a “Pass” and indicates clear task completion with no hint, clarification, or reminder required. One means “Help” and indicates one hint was provided for the task to be completed. Zero means “Fail” and indicates the task could not be completed despite providing the participant with two hints or more. Task-related usability issues occurred with an average UER of 1.1. For more depth regarding this data, see Additional file 2 which analyzes the data. The same usability issues were analyzed using a percentage pass rate.

**Table 2:** Use error rating definitions, shown as nominal and numerical codes

Normative Use Error Rating	Numerical Use Error Rating	Definition	
Pass	2	User completed task with no hint, clarification, or reminder	
Help	1	User completed task with one hint	
Fail	0	User did not complete task despite several hints	

To ensure appropriate evaluation, the 20 types of tasks attempted were coded by two raters (authors YL and JT). Interrater reliability was reported both as an absolute percent agreement and equal weighting Cohen’s Kappa, taking into account chance agreement [32]. Where there was disagreement, agreement was reached through discussion. Based on the associated numerical code for each clinical group, an average UER was calculated for each task. The average UER was also calculated for each group of tasks which represented the three general functions of the software (e.g. tracking, trajectory, and triggering). Finally, a global UER average was calculated for all participants and for all tasks.

#### Usability issue severity level

Potential severity of the use error was categorized as minor, if patient was unlikely to be harmed; moderate, if patient could be temporarily harmed; or high, if patient could be permanently harmed. This was coded by one rater (YL) and confirmed with an expert physician intensivist (author AMG). In the case of discrepancies, final score was determined through discussion. This approach was used to rate the importance of a use error [33].

## Results

### Participants

At the time of the study, the 22 participants were full-time ICU staff. Participant demographics are shown in Table 3. From the pre-session questionnaire, only 27% of participants received formal training when it was offered over two years ago. Though 64% of the participants were aware of the software, 82% rarely or never used.

**Table 3:** Demographics, clinician specialization, training, current use, and awareness of data integration software

		Physicians	Nurses	Respiratory Therapists	Global Proportion
Total Number		7	8	7	22
Gender, % (n)	Male	14 (*n* = 1)	-	14 (*n* = 1)	9 (*n* = 2)
	Female	86 (*n* = 6)	100 (*n* = 8)	86 (*n* = 6)	91 (*n* = 20)
ICU Experience, % (n)	<1 year	43 (*n* = 3)	13 (*n* = 1)	29 (*n* = 2)	27 (*n* = 6)
	1–3 years	29 (*n* = 2)	25 (*n* = 2)	-	18 (*n* = 4)
	4–10 years	29 (*n* = 2)	25 (*n* = 2)	57 (*n* = 4)	36 (*n* = 8)
	>10 years	-	38 (*n* = 3)	14 (*n* = 1)	18 (*n* = 4)
ICU Shifts/Week, % (n)	1–2 times/week	-	25 (*n* = 2)	29 (*n* = 2)	18 (*n* = 4)
	3–4 times/week	29 (*n* = 2)	75 (*n* = 6)	71 (*n* = 5)	59 (*n* = 13)
	>4 times/week	71 (*n* = 5)	-	-	23 (*n* = 5)
ICU Specialization, % (n)	CCCU^a^	29 (*n* = 2)	63 (*n* = 5)	-	32 (*n* = 7)
	PICU^b^	29 (*n* = 2)	38 (*n* = 3)	-	23 (*n* = 5)
	PICU/CCCU	43 (*n* = 3)	-	100 (*n* = 7)	45 (*n* = 10)
Previous Training with Software, % (n)	Yes	14 (*n* = 1)	50 (*n* = 4)	14 (*n* = 1)	27 (*n* = 6)
	No	86 (*n* = 6)	50 (*n* = 4)	86 (*n* = 6)	73 (*n* = 16)
Software Use/Shift, % (n)	Several times/shift	29 (*n* = 2)	-	-	9 (*n* = 2)
	Once/shift	14 (*n* = 1)	13 (*n* = 1)	-	9 (*n* = 2)
	Rarely during a shift	43 (*n* = 3)	-	-	14 (*n* = 3)
	Never	14 (*n* = 1)	88 (*n* = 7)	100 (*n* = 7)	68 (*n* = 15)
Awareness of Software, % (n)	Yes	71 (*n* = 5)	75 (*n* = 6)	43 (*n* = 3)	64 (*n* = 14)
	No	29 (*n* = 2)	25 (*n* = 2)	57 (*n* = 4)	36 (*n* = 8)

^a^CCCU: Cardiac critical care unit; ^b^PICU: pediatric intensive care unit

The extent of underuse was unknown when usability testing was carried out. Consequently, the pre-session questionnaire did not ask participants why they did not use the software. The research team included the question “Did you know that T3™ is accessible from all PC workstations?” because they suspected that staff were unaware they had access to the software. Of the 20 participants who answered this question, 14, or 70%, were aware they could access the software. Two participants, who knew they had access but did not use T3™, provided insight as to why they did not use it. One nurse preferred to look at the physiological monitor because it offered a real-time view of the patient status with more detail than T3™. The other nurse stated it could compliment his/her view of the patient status if s/he had time to use it during his/her shift. These findings suggest that, at the very least, most participants did not extensively use the T3™ software and were naïve to the software.

### Interrater reliability

For all attempted tasks by each participant, the interrater reliability of the UER was 89% between the two raters (YL and JT). This is in absolute agreement with an equal weighted Cohen’s kappa of 0.85, corresponding to a strong level of agreement [32].

### Software strengths (aid to task completion) and usability issues (hindrance to task completion)

#### Overview

**Fig. 4:**
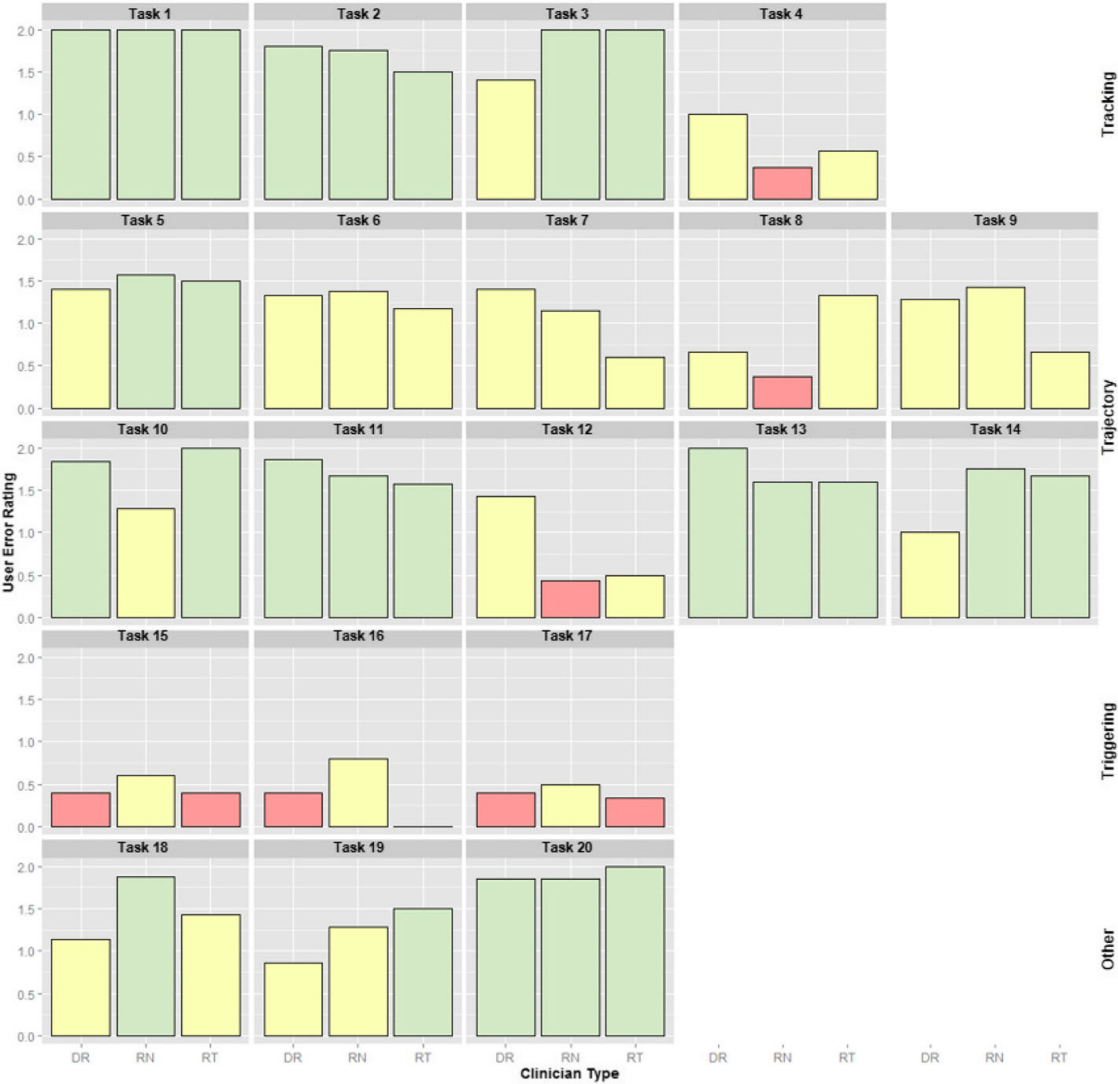
Variation of use error ratings across clinician disciplines for all tasks related to tracking, trajectory, and triggering as well as other software functions. Three levels of use error ratings (UERs) were employed by two raters and averaged for each type of clinician for 20 tasks. The UER distribution was further grouped by function: tracking (Tasks 1–4), trajectory (Tasks 5–14), triggering (Tasks 15–17), and other (Tasks 18–20). Usability issues, defined as tasks with a UER of 1 or less and highlighted in yellow or red, were dependent on the type of task and, to a lesser extent, on the type of clinician. Most usability issues were centered on the trajectory and triggering functions. UER: Pass (P) = 2 (*green*), Help (H) = 1 (*yellow*) and Fail (F) = 0 (*pink*). Clinician groups: DR: physician intensivists, RN: intensive care nurses, and RT: respiratory therapists

Due to time constraints, not all 20 types of tasks could be completed. Participants attempted an average of 18 of all 20 types of tasks (88%). The task groups representing the three main software functions had the following UER: 1.5/2, or “Pass”, for tracking; 1.3/2, or “Help”, for trajectory; and 0.4/2, or “Fail”, for triggering. For all tasks, the overall UER was similar across disciplines with a UER of 1.3 for physicians, a UER of 1.3 for nurses, and a UER of 1.2 for respiratory therapists. A summary of the average ratings, by tasks and clinician groups, are shown in Table 4 and illustrated in Fig. 4.

**Table 4:** Usability tasks tested with severity levels and use error ratings

General Functions	Tasks Tested for Each Function	Error Severity Level	Average Use Error Rating by Task and by Clinician Type			Average Use Error Rating by Task
			Physicians (*n* = 7)	Nurses (*n* = 8)	Respiratory Therapists (*n* = 7)	
Tracking: Orientation (4 tasks)	1. Locating patient file	High	P (2.0)	P (2.0)	P (2.0)	P (2.0)
	2. Identifying a value for a specific physiological variable	High	P (1.8)	P (1.8)	P (1.5)	P (1.7)
	3. Estimating duration of event by identifying two time points	High	H (1.4)	P (2.0)	P (2.0)	P (1.8)
	4. Manipulating time scale	High	H (1.0)	F (0.4)	H (0.6)	H (0.6)
	Function Use Error Rating by Clinician Type		P (1.5)	H (1.5)	P (1.5)	P (1.5)
Trajectory: Relationships between Parameters (10 tasks)	5. Comparing trends for two specific parameters	High	H (1.4)	P (1.6)	P (1.5)	H (1.5)
	6. Comparing different patient physiological states	High	H (1.3)	H (1.4)	H (1.2)	H (1.3)
	7. Identifying values for two specific parameters at an event	High	H (1.4)	H (1.1)	H (0.6)	H (1.0)
	8. Identifying vital signs (group of parameters) prior to an event	High	H (0.7)	F (0.4)	H (1.3)	H (0.8)
	9. Viewing trend of three redundant overlapping parameters	High	H (1.3)	H (1.4)	H (0.7)	H (1.1)
	10. Viewing infusion medication data	High	P (1.8)	H (1.3)	P (2.0)	P (1.7)
	11. Comparing infusion medications with vital signs	High	P (1.9)	P (1.7)	P (1.6)	P (1.7)
	12. Detecting change in infusion medication rate over time	High	H (1.4)	F (0.4)	H (0.5)	H (0.8)
	13. Viewing ventilator data	High	P (2.0)	P (1.6)	P (1.6)	P (1.7)
	14. Viewing laboratory data	High	H (1.0)	P (1.8)	P (1.7)	H (1.5)
	Function Use Error Rating by Clinician Type		H (1.4)	H (1.3)	H (1.3)	H (1.3)
Triggering: Automated Integration (3 tasks)	15. Viewing target ranges using shading (semi-automatic aid)	Moderate	F (0.4)	H (0.6)	F (0.4)	F (0.5)
	16. Sparkline (automatic trend line for one variable)	Minor	F (0.4)	H (0.8)	F (0.0)	F (0.6)
	17. IDO_2_ indicator (automatic computation using 16 parameters)	High	F (0.4)	H (0.5)	F (0.3)	F (0.4)
	Function Use Error Rating by Clinician Type		F (0.4)	H (0.6)	F (0.2)	F (0.4)
Other Functions (3 tasks)	18. Finding notes	High	H (1.1)	P (1.9)	H (1.4)	H (1.5)
	19. Modifying/adding note	Moderate	H (0.9)	H (1.3)	P (1.5)	H (1.2)
	20. Setting targets	Moderate	P (1.9)	P (1.9)	P (2.0)	P (1.9)
	Function Use Error Rating by Clinician Type		H (1.3)	P (1.7)	P (1.6)	P (1.5)
All functions	Global Function Use Error Rating, for All Functions by Clinician Type and for All Clinicians		H (1.3)	H (1.3)	H (1.2)	H (1.2)

(Minor, Moderate or High) (Pass (P) = 2, Help (H) = 1 and Fail (F) = 0)

#### Tracking function

Tracking describes the general function of patient census navigation (using the dedicated census page or short-cut drop-down menu) and time orientation (using time series visual aids). It is a critical function since making time-sensitive decisions on the wrong patient, or with data that corresponds to a mistaken time period, can potentially lead to patient harm. The average UER for patient tracking tasks was 1.8, indicating that participants completed tasks with little or no help. All tracking tasks had potentially high clinical impact severity. Clinicians easily completed three of four patient tracking tasks: Task 1) locating a patient in the census, Task 2) identifying a value for a specific physiological variable, and Task 3) estimating duration of an event by identifying two time points. However, clinicians had difficulty completing Task 4) manipulating the timeline, which corresponded to a UER of 0.6.

##### Tracking usability issue: Situating the patient data in time

Though clinicians could choose their patient and select data from a given time period of data, they could not easily select specific time periods. To test participants’ ability to situate the data in time, participants were asked to determine the patient’s length of stay by manipulating the interface from a default view showing partial patient data. All clinician groups encountered difficulty with this task, demonstrated by UER scores of 1.0 for physicians, 0.4 for nurses, and 0.6 for respiratory therapists.

**Fig. 5:**
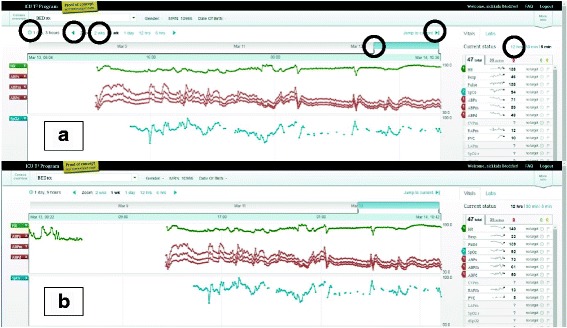
Usability issue of time manipulation interface. Screenshots of patient view with time manipulation interactive light blue icons, circled on top section, with heart rate, arterial blood pressures, and oxygen saturation data streams. Screenshot **a** appears to show start and end of data but screenshot **b**) shows the same gap as an interruption in the data streams, signifying the patient was away from the ICU and therefore, was not continuously monitored

This task can be parsed into three successive steps: 1) condense all the collected data into a single window, 2) check the start and end of the data, and 3) mentally calculate the entire length of stay. Task difficulty may be due to the first two steps which required clinicians to understand how to use the six interactive features for time manipulation (See circles in Fig. 5a). Clinicians needed to manipulate the interface and find the start and end of the patient data. Since this was a “live” patient, the start and end of the data indicated to clinicians when continuous monitoring of the patient started and, consequently, when they first came to the ICU. The six interactive features were, at times, imperceptible to participants and required high visual acuity, as well as manual dexterity. As participants looked back at the parametric data in time, they assumed they had found the start of the patient data if they encountered a gap (See Fig. 5a). When prompted to continue to look back, they found that there was still more data (See Fig. 5b). These two screenshots show how the interface did not communicate to clinicians the start and end of patient data and could leave users with a sense of uncertainty about whether they were seeing all the data for a particular patient.

In conclusion, for the tracking function, most clinician groups could complete three of four tasks, but the main usability issue centered on the task requiring precise and accurate manipulation of data presented as a time series. For the specific task of viewing all the data for a particular patient, exploring the data may leave users with a sense of uncertainty or frustration. Some participants asked for a manual input of the horizontal (time) range suggesting they did not feel they could choose the time window of data to a satisfying extent.

#### Trajectory function

**Fig. 6:**
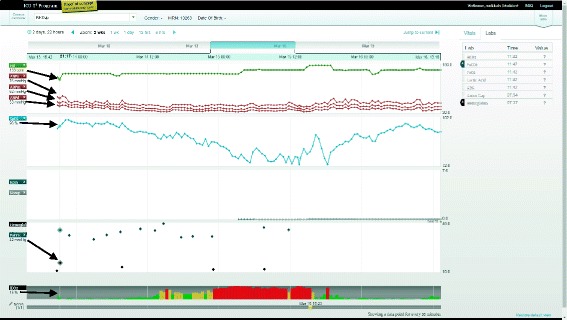
Time series data visualization of multiple physiological signals and therapeutic interventions. Screenshot of patient view showing four view panels with data streams for heart rate and arterial blood pressures in the *top panel*; oxygen saturation in the second from *top panel*; medical infusions for epinephrine and norepinephrine in the third from top panel; and blood gas analyses for hemoglobin and carbon dioxide partial pressure in the *bottom panel*. The identified values for March 13th at 21:17 are found at the left-hand side of the screen and are related to the point in the time series by *arrows*

Clinicians closely monitor patient trajectory for rapid or gradual changes by comparing current physiological monitor data to daily target thresholds. With the availability of continuous data from a patient’s entire ICU stay, determining trajectory then involves viewing related parameters and investigating both overall trends and point data. To support such analysis, we asked clinicians to complete ten tasks (Tasks 5 to 14), which tested how easily clinicians could create multiparametric visualizations (Tasks 5, 6, 9, 10, 11, 13 and 14) and extract data from these complex visualizations (Tasks 7, 8 and 12). Creating multiparametric visualizations required clinicians to intuitively understand how to select parameters from a list and view them together on one of four panels (See Fig. 6). Identifying a single point of data required clinicians to hone in on the time series visualization and read off the chosen parameter’s value on the left-hand side (See Fig. 6). Of the ten trajectory tasks, clinicians failed to complete four (Tasks 7, 8, 9 and 12) and required little or no help (an average UER above 1) to complete the remaining six tasks (Task 5, 6, 10, 11, 13, and 14) (See Fig. 4 and Table 4). All clinician groups had similar UERs for this set of tasks with 1.4, 1.3, and 1.2 for physicians, nurses, and respiratory therapists, respectively (See Table 4).

##### Trajectory software strength: Creating multiple parametric visualizations

Generally, seven tasks (Tasks 5, 6, 9, 10, 11, 13, and 14) were used to test how clinicians used the software to visualize multiple parameter trends. Essentially, the tasks were to find parameters and add them to a default of three basic vitals: heart rate; systolic, diastolic, and mean blood pressures; and oxygen saturation. Task completion generally had a good UER above 1.3, except for Task 9 which had a UER of 1.1 due to unfamiliar data labels assigned at a different ICU. Physicians, nurses, and respiratory therapists required little or no facilitation to accomplish the task of combining different parameters (Task 5, 6, 10, 11, 13 and 14), and the combined average of all three groups was above 1.0 when creating complex visualizations.

Task 11 was used to test how easily clinicians could visualize both intervention and physiological data streams, thereby, investigating their interrelationships. Most clinicians successfully completed this task and had a group UER of 1.9, 1.7, and 1.6 for physicians, nurses, and respiratory therapists, respectively. One nurse stated that instead of looking at infusions and vitals separately, making it necessary to recall a child’s baseline physiological vitals from memory, the software supported this task by displaying both types of parameters on the same graph. A second nurse remarked that it was “easier to put together the picture [compared to the current electronic charting system]” and, similarly, one physician remarked “I’m not working as hard with T3™ to make a mental visualization”. These comments indicate that participants liked how the software helped them to visualize parameter trends or see all the pieces of the puzzle.

##### Trajectory usability issues: Using multiple parametric visualizations

Intensive care requires knowledge of both overall patient trajectory, spanning their ICU stay, and the immediate trajectory, such as in response to a therapeutic intervention. Software should partially off-load the cognitive processes required to transform numerical, short-term data into longitudinal trends without losing the granularity of the point data. In this study, once clinicians chose and viewed a set of parameters from dozens available, they were asked to extract and understand nuances about the combined trends. Two types of tasks tested how clinicians interpreted multiparametric visualization: 1) identifying point data (Tasks 7 and 8), and 2) detecting change (Task 12).

To hone in on the time of an event, both Tasks 7 and 8 required time manipulation, a core usability issue previously discussed. Participants were asked to report values for parameters by identifying point data from the trends. This dynamic manipulation of the interface required high visual acuity, manual dexterity, and visual sensitivity to display data for a given time period. It also required the ability to scan values associated with each parameter chosen (See Fig. 6). Clinicians had more difficulty reporting values for groups of parameters (Task 8) than two specific parameters (Task 7).

**Fig. 7:**
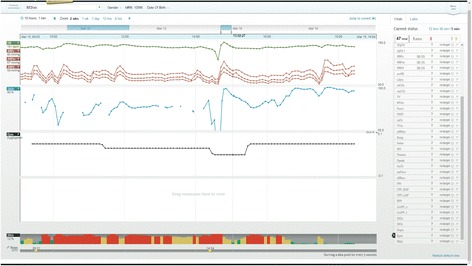
Usability issue of auto-fit scaling resulting in misinterpretation of when the medical infusion ceased. Screenshot of epinephrine infusion with auto-fit scaling resulting in a negative infusion rate of −0.1 μg/kg/min and a rate of 0 μg/kg/min plotted as a line in the middle of the graph area

Task 12 required clinicians to detect when a continuous infusion was stopped. Though physicians (a task average UER of 1.4) could better detect an interruption in the infusion than nurses (a task average UER of 0.4) and respiratory therapists (a task average UER of 0.5), most participants failed to notice this. This may be due to 1) an infusion rate of 0 μg/kg/min was plotted as a continuous line, and/or 2) the automatic vertical scaling feature called “best-fit” created a vertical range of −0.1 to +0.1 μg/kg/min (See Fig. 7). Participants were often surprised that a rate of 0 μg/kg/min was plotted as a line in the middle of the graph and, instead, expected a gap in the data when the rate was 0 μg/kg/min. A higher physician UER may also be explained by the investigative nature of physician work, more advanced training in pharmacokinetics, and their role as initiators of medical infusions.

The failure to detect change could be attributed to distraction from the multiple viewing panels (four) that were populated by several parameters of different scales and may have divided participant attention, making detecting parameter changes more challenging. Furthermore, detecting change only from the time series pattern may have been troublesome due to a small font size.

Participants suggested scaling based on realistic parameter ranges. For example, medication infusion scales should always start from 0 since negative infusion rates are impossible and differences in orders of magnitude between infusions should be graphed as to not dwarf each other (e.g. dopamine and epinephrine differ by two orders of magnitude). Additionally, scales for temperature plots should start at approximately normal body temperatures to help highlight important variances around the baseline to be more informative than if the scale started from 0.

#### Triggering function

Currently, monitoring a patient involves data from physiological monitors displayed as short-term (e.g. below a minute) waveforms and numerical values with visual or audible alarms to signal out-of-range targets. To partially off-load the cognitive processing of monitoring, the software provided three visual aids, or triggers to decision-making, which are overlaid on the long-term time series data to make unstable time periods more apparent. The triggers were either semi-automated, requiring clinician input, or fully-automated visualizations, derived only from the data. Deviations from baselines were highlighted by 1) shading time series data, 2) displaying mini-trends (sparklines) with exaggerated minima and maxima, and 3) by automatically computing and displaying the risk of inadequate oxygen delivery (IDO_2_) as a color-coded bar graph (See Fig. 3). Thus, the software highlighted periods of continuous data with undesirable trajectory, either for single or combinations of parameters. In this way, clinicians may interpret data faster by focusing their attention on a portion of data from the computer-generated visual trends instead of memorizing and creating their own long-term mental trends.

To test the triggering function, clinicians were asked to use the visual aids of shading (Task 15), sparklines (Task 16), and the IDO_2_ indicator (Task 17). In general, participants ignored the visual aids until they were asked to attempt the task and all had UERs below 1, with a global triggering UER, aggregated by task and clinician type, of 0.4 (See Table 4). Specifically, one physician’s comment on shading out-of-range values: “I'm not sure if the shading is helpful, I'm getting distracted by the area under the curve or the shape or something. Maybe if target ranges were shown as two straight lines across the graph.” Clinicians stated that sparklines did not provide enough detail to be useful. One nurse commented that “[they] prefer[red] just looking at the graph than looking at that little [graph] on the side because it's bigger, you can see [the graph] better.” Usability of the IDO_2_ trigger will be discussed in following section.

##### Data reduction: IDO_2_ indicator

The IDO_2_ indicator, derived using a 16-parameter algorithm, calculates and displays the risk of inadequate oxygen delivery. Six out of seven physicians were unaware of the IDO_2_ indicator and were skeptical of it because they did not know how it was derived. One physician’s mistrust was voiced as follows: “I don’t believe this [indicator] because I don’t know where it came from [or] what formula [it is based on].” In addition, since physicians regularly integrate data and derive their own assessment of patient instability, they stated that the indicator was redundant with their own assessment. For physicians using IDO_2_ for the first time, the indicator did not provide enough predictive value for them to incorporate it into their clinical practice. As one remarked “It’s almost too late. [IDO_2_] shows you when they are unstable rather than trying to predict adverse events. [IDO_2_] tells me what I already know.” However, one physician who had prior knowledge of the IDO_2_ indicator and trusted the underlying algorithm remarked that it would prompt investigation and “pull in more variables to explain what was seen [as a period of instability].”

Nurse impressions of IDO_2_ were mixed with some voicing confusion as to the meaning of the indicator as well as annoyance, and others voicing usefulness to confirm their own assessment. As one nurse stated "I don't really know what this graph is trying to tell me. If it's just telling me that the [saturations] are low, I already know that the [saturations] are low." Though all nurses found a correlation between the IDO_2_ indicator and their own assessment, made directly from vitals data, they disagreed on whether this was advantageous or redundant. Similar to the physician group, nurses were skeptical of the new indicator because its derivation was unclear.

Respiratory therapists found that the indicator correlated with their assessment of instability from the hemodynamic data and also indicated that they would use it if they could trust it. As one respiratory therapist stated, "because I don't know how this percentage is calculated or what it takes into account, I don’t find that it is useful other than the color coding which is very intuitive." Respiratory therapists stated the indicator could help them assess quickly and prompt further investigation: “[IDO_2_ is] a first look at what’s going on. If you want details you can look at parameters more closely”; “it only really tells me that I need more information”; and "[it] might prompt me to be proactive about suggesting different modalities. Especially because it's O_2_-related, it would prompt me as an RT to think outside the box." In summation, the IDO_2_ indicator may potentially help clinicians proactively detect deterioration, but the software should allow users to understand how it was derived.

Visually, when attention was called to the persistent bar graph of the IDO_2_ indicator participants all interpreted it correctly with higher values represented as red bars thus perceiving the patient as being at high risk of inadequate oxygenation. This composite parameter could also be seen as a time series on one of the four graph areas. Two physicians, one nurse, and one respiratory therapist viewed the IDO_2_ indicator as a time series. One physician found that the indicator correlated well with the charted events while the respiratory therapist found that it correlated with the hemodynamic data. The nurse preferred the bar graph visualization to the time series. One important difference between the bar graph and the time series was that each bar was color coded with low IDO_2_ values in green, intermediate values in yellow, and high values in red.

#### Other functions: Charting

In general, clinicians could easily use the charting features of the software. UERs were high for physicians, nurses, and respiratory therapists with 1.3, 1.7, and 1.6, respectively (See Table 4). All clinician groups could easily set targets (Task 20) and visually highlight out-of-target values for a given parameter trend line but were less able to find and write notes (Task 18 and 19).

#### Summary of results

In summary, for all functions and tasks the UERs were similar across clinician groups with physicians, nurses, and respiratory therapists having a global UER of 1.3, 1.3, and 1.2, respectively, indicating a need for some facilitation. The UER for tracking, trajectory, and triggering functions for all users and groups of tasks was 1.5, 1.3, and 0.4, respectively. The main tracking usability issue was time manipulation while the main trajectory usability issues were identifying multiple data points and detecting changes in the data. All three triggering aids had usability issues due mainly to a lack of transparency about who set the target ranges and what parameters contributed to the calculation algorithm.

Despite usability issues, it is undeniable that the dynamically displayed time series visualization and complimentary algorithms helped clinicians visualize and interpret the many overwhelming high-frequency data streams. Testing these novel data visualizations is a first step at observing how computerized pre-processing of data can be communicated to clinicians in realistic scenarios and for tasks supporting clinical decision-making.

## Discussion

Usability testing revealed how data integration software supported or hindered tasks that require use of continuous patient data by a representative sample of end users. The qualitative nature of our study provided insight into the user experience and opportunities for user-centered design modifications. Clinicians had a high degree of flexibility and, consequently, easily produced data dense visualizations but encountered usability issues of time manipulation, point data identification, and detection of trend deviations. These issues confirm those identified using the heuristic evaluation method [22]. Attributable themes include the transformation of point data into time series visualization, the emergence of visual pattern overload, visual aids representing computer-processed data, data trustworthiness, and use variability among clinical disciplines.

### Transforming numerical point data to long-term, time-scaled visualizations

Through tracking and trajectory tasks, we found that time series visualizations were appreciated by clinicians since it off-loaded their existing cognitive task of creating visualizations from continuous numerical patient data. This may indicate that the software alleviated point data overload. Point data recall was effective for single parameter trends. However, multiparametric visualizations lead to denser and overlapping time series making the recall of multiple point data difficult. While some confusion and misinterpretation was observed, we found that time series data displays allowed for quick determination of the duration of instability. The software provided clinicians with a high degree of choice and flexibility to create multiparametric visualizations. However, it consequently limited their ability to interpret and extract point data. The effort required to make distinctions between the alternatives appear to outweigh the benefits of having many options and is consistent with Schwartz’s statement in “The Paradox of Choice”: “choice no longer liberates, but debilitates” [34]. A suggestion for improvement is to simplify the user interface by eliminating some interactive features and communicating to the user the meaning of each feature to facilitate clear action, to reduce confusion, and to make the user feel in control of the system.

As previously mentioned, manipulating the software interface required high visual acuity and manual dexterity causing tasks to be somewhat time consuming. In addition, clinicians were unaccustomed to the large choice of continuous parameters. Consequently, participants voiced frustration after completing tasks because they expected to have completed them much faster. This is consistent with Hick’s law which postulates that time on a task is positively correlated with number and complexity of choices, and as time to decision increases user satisfaction decreases [35]. This further reinforces that if the interface has poor usability, then low uptake may result since real ICU tasks are highly time-sensitive.

### Integrating data trends: Visual pattern overload

The availability of dozens of data streams on a single software platform is an undeniable advantage over existing dispersed clinical information systems and is a crucial step to understanding relationships between parameters [36, 37]. The software helped clinicians visualize multiple parameters as a time series on a single chart. This represented thousands or millions of data points but were boiled down to single patterns which resulted in dense visualizations. For a given parameter, individual data points were transformed to more discernable patterns. However, when participants combined multiple parameters we observed a phenomenon of “visual pattern overload”. Consequently, participants experienced difficulties in extracting specific data or detecting subtle changes among the many patterns. To address the usability issues associated with multiparametric trends, we suggest four strategies outlined below to better support their use.

#### Pre-defined parametric grouping

The need for integrating technologies to show relationships between parameters is a paramount function of data integration software [36, 37]. As Feyen stated, “It is not the monitoring that makes the difference but how this is translated into more appropriate and targeted treatments” [38]. Since interventions act on groups of physiological parameters, visual clutter may be reduced if displays are reassembled according to intervention, helping to inform targeted treatments. For example, dopamine infusions can be automatically grouped together with heart rate and blood pressure, upon which they are known to act. Similarly, mechanical ventilation, which acts on pulmonary physiological parameters, could be grouped with peripheral oxygen saturation and carbon dioxide data. Parameter grouping through configurable displays has been studied for basic vitals [39]. However, with more advanced monitoring modalities, further systematic selection of parameters is warranted for each medical infusion or organ support data stream. Hajdukiewicz et al. postulated the use of the abstraction hierarchy (AH) framework to represent the patient data and information at several levels of aggregation and abstraction [40]. This framework supports problem-solving and embodying the current state of biomedical knowledge [40, 41]. For interface designers, AH patient representations offer a means of allocating roles and responsibilities to different clinical specialties. Also, it structures data from monitoring devices and therapeutic interventions by mapping the types of data onto the patient model, at defined levels of abstraction and aggregation. In this way, configurable displays may off-load the task of selecting relevant parameters and minimize superfluous data streams. Thus, the clinician is aided in determining cause-and-effect relationships and supported in their problem-solving activities.

#### Scaling according to the nature of the parameters

The issue of automatic scaling was provided as a “one size fits all” solution; however, clinical parameters have known limitations anchored in the use of the medical devices or knowledge of human physiology. A few examples are that medical infusions cannot be negative, differences in infusion rates vary by orders of magnitude, and the temperature of the living human body generally stays within a few degrees of baseline. Inappropriate scaling led clinicians to ignore parametric changes or created mistrust of the software. Usability testing incited clinicians to describe appropriate and realistic scaling for different types of parameters. These preferences could be easily programmed into the software, avoiding or minimizing the false conclusions observed during testing. Therefore, usability testing was instrumental in highlighting how data dense visualizations can be confused and, consequently, be rectified in a subsequent software iteration.

#### Data reduction using algorithms

Visual pattern overload reduction and pre-defined parametric grouping were automatically performed through the IDO_2_ algorithm. The percentage risk of inadequate oxygen delivery was displayed as a persistent bar graph at the bottom of the screen. In this way, data for 16 parameters were effectively reduced and was intuitive to understand. Now, the physician’s complex cognitive process of relating respiratory physiology and medical interventions to gauge oxygen delivery, typically from disparate monitors, can be off-loaded. In addition, IDO_2_’s estimation of risk addresses the problem of uncertainty inherent to the dynamic nature of critical care and supports the high-level analytical task of decision-making [42, 43].

Lack of transparency and published evidence of the new composite IDO_2_ parameter led to mistrust and was the main barrier to its use. The only clinician familiar with it was prompted to investigate further. Thus, our findings suggest that although triggering functions such as the IDO_2_ indicator have the potential to aid with patient monitoring, it is imperative that the interface communicate how new indicators were derived. As an early warning system, the IDO_2_ indicator could achieve what Bion described as the proactive identification of early changes to “empower ward staff to call for help and initiate further investigation to prevent or limit the magnitude of adverse events” [44]. Observations from usability testing warn that without consistent exposure and integration into clinical practice, data interpretation aids may be ignored, and, thus, excluded from critical decision-making where they would be most useful.

#### Novel visualizations

In our study, we tested four types of visualizations and found that time series visualization worked well for single parameters but was less usable when parameters were combined. In addition, highlighting out-of-target range data, using shading, as well as exaggerating minima and maxima by using sparklines were imperceptible to participants. Tasks that required specific use of multiparametric data should be developed to further test these and other types of data-dense visualizations. For example, metaphor displays that use various shapes to represent physiological processes have been explored in anesthesia [45]. Indeed, Doig suggested using shapes to help nurses better visualize hemodynamic parameters [37].

### Data trustworthiness

The integrated single-view of multiple data streams improved the trustworthiness of the data as a whole. For example, by viewing both etCO_2_ data and intermittent CO_2_ blood gas data, respiratory therapists could confirm and trust the continuous etCO_2_ trend. Also, continuous data streams complemented the event notes and may benefit charted notes on the electronic medical record (EMR). For example, when ventilator pressure drops to 0 mmHg, respiratory therapists could assume this was the exact time the ventilator was disconnected and manual bagging was initiated. Indeed, Doig found that to prevent data from going unused, it was necessary to contextualize data [37]. Redundant data and additional clinical context may improve data trustworthiness of continuous data itself and the charted patient record. Future clinical information systems should integrate MMM data with EMR qualitative information to provide a complete picture of the patient and automatically check data integrity.

### Usability testing with diverse clinician groups

Bion states that information technologies require “staged “bottom-up” development, pilot testing, and appropriate implementation into existing hospital culture” [44]. Given the complexity of intensive care and the high degree of specialization of the critical care professional, feedback from representative end-users is essential for acceptance of the software. This study provides recommendations for appropriate implementation by revealing aspects of the ICU culture that would impact software acceptance.

Different types of clinicians required different levels of data granularity. Physicians operated on a longer patient timeline than nurses, who usually operate within seconds or minutes, and respiratory therapists, who usually operate on a moderate timeline. Therefore, averaged values, over five seconds, were not as useful to nurses but were more usable to physicians and respiratory therapists. To encourage system usage with nurses, more precise data should be made available. To support appropriate decision-making, the display should show preferred data streams for each clinical specialty.

### Proposed iteration and improvements

At the time of writing, a new version of the software, which addressed heuristically found usability issues, was launched. This new version improved the reading of values on the time series trend by displaying the changing value close to the scanning cursor, as well as its font size and style. Also, absolute maximum and minima of the trend is now always visible. In addition, shortcuts for viewing grouped parameters related to respiratory or hemodynamic functions are now available. This study’s usability issues should be addressed if the software is to be useful to clinicians. Future iterations should offer support to select, filter, reduce redundant data streams, provide contextual meaning to the data, and provide novel visualizations that are intuitively understood. For example, pairing etCO_2_ with pCO_2_, which respiratory therapists do to determine trustworthiness of the etCO_2_ continuous trend can be readily available if a respiratory therapist is detected as the user. Better still, employing algorithms to correct the etCO_2_ trend using more reliable pCO_2_ blood gas values and eliminating redundant data thereby reducing overall data and pattern overload. In the long-term, the future versions of the software should integrate with the medical record system or new medical record systems should integrate with the existing data visualization software so as to provide a single source of patient data and information. Table 5 provides practical suggestions for data integration and visualization software.

**Table 5:** Practical Improvement Suggestions for Data Integration and Visualization Software

Improvement	Rationale	Suggestions to Achieve Improvement
Reduce redundant data streams.	Removal of redundant data is required to allow clinicians to efficiently and easily abstract, trend, and interact with the data.	Ensure preprocessing mass volumes of continuous real-time data. For example, employ algorithms that corrects etCO_2_trends using pCO_2_ blood gas values.
Provide user awareness.	User-aware applications that dynamically adjust the data display mode based on the user context can ensure that adequate and relevant data needs are being displayed and enhance clinicians’ efficiency and efficacy in extracting meaningful information.	Provide customized view of patient data tailored to the clinician’s needs. For example, if a respiratory therapist is detected as the user, the system would display etCO_2_ with pCO_2_ to help respiratory therapist know if s/he should trust the etCO_2_ continuous trend data.
Reduce clinician cognitive demand in interacting with the visual displays.	Ensuring that components that are important for decision-making are represented in the display in a perceptually similar manner as to improve the clinician’s decision-making accuracy and efficiency.	Present the components that are important for decision-making as an integrated object and/or by presenting them close together spatially or temporally.
Mandate integration of data integration and visualization software with existing medical record systems.	Integration of data integration and visualization software with medical record systems to provide a single source of patient data which facilitates data synchronization and may reduce use errors.	Technology procurement policies should require incoming data platforms to freely exchange data and information with existing clinical information systems.
Provide easy time navigation.	A critical function of the interface is enabling the user to rapidly select the time frame of continuous data, relative to the patient’s stay in the ICU.	Provide interface controls which support both exploratory data navigation across time and specific user defined timeframes.
Ensure interface is flexible to different types of users and levels of expertise.	Functions which are learned should provide shortcuts for accelerated performance.	Provide layered function description and interface shortcuts.
Ensure software responsiveness.	Additional data streams and access to denser data visualizations may slow down system performance and diminish user satisfaction and decision-making quality.	Ensure new data streams are compressed or back-end processing is sufficient to maintain adequate responsiveness.

Although this study focused on the usability of data integration software, pre- and post-session questionnaires, the think-aloud nature of the test method revealed aspects of clinical work which may explain software underuse in clinical practice. For example, the pre-session questionnaire indicated that six of the 22 participants received training. Training was provided when the software was launched in the unit but was not mandatory and was not provided on an ongoing basis to incoming staff. In addition, nurses and respiratory therapists dedicate a large proportion of their time to charting on the medical record accessed from the bedside computer terminal. Since the software was web-based, it required clinicians to stop charting, pull up the web-browser and login on the computer they use to chart. Therefore, staff may deprioritize accessing the data visualization software because of the numerous steps required to do so. In addition, the unit’s UNIX-based EMR system was replaced by a Windows-based system during the study period. Staff may also have devoted more time to learning the new EMR system and had even less time to explore auxiliary data platforms such as T3™.

In the end, our work indicates that the ideal system for capturing and utilizing continuous physiologic date in the intensive care unit will allow seamless integration into work flow, is intuitive and fits with the way clinicians think and work, and is trusted as a platform that diminishes work and enhances decision-making, rather than contribute to additional confusion, uncertainty, or skepticism.

### Improvements over existing work

While planning the overall project, we carried out a systematic review on data integration and visualization software, finding nine studies reporting on level of usability and satisfaction by means of a questionnaire and scale [29, 39, 46–52]. These studies report usability as a system global characteristic measured on a continuous scale and was self-reported by participant. Another study by Peute et al., reported the change in the number and type of usability issues following user-centered design changes [53]. These studies did not relate issues to specific clinician tasks or software interface features. Our study assessed usability based on tasks, users, and software features to provide tangible suggestions to improve software design of interfaces running on similar computational hardware and operating platforms.

We identified factors influencing usability of a fully interactive, commercial data integration system by physicians, nurses, and respiratory therapists. Also, usability testing enabled a deeper understanding of how continuous data was identified and interpreted by three distinct clinical specialties. Finally, recommendations for future iterations of the current software were provided as well as a description of an ideal integrated data and visualization software platform.

### Limitations

The software we tested addressed several theoretical informatics barriers and our findings may be generally applied to software with a similar level of data integration. Our simulations tested how untrained participants used the software and provided insight as to the intuitiveness and ease of the basic tracking functions. A training session focused on the tracking function tasks may have aided the use of trajectory and triggering functions. Future usability testing could include training and focus on tasks related to higher-level visualization functions.

The simulations tested 20 types of tasks, most of which were explorative in nature and more closely related to physician work than the other occupations. As such, these simulations forced nurses and respiratory therapists to perform investigative tasks outside their usual work scope. In reality, they spend more time charting or working directly with the patient or other monitoring devices. Any new software should aim to integrate the data from these technologies and reduce the burden of charting if it is to be useful to these groups of users.

The simulation environment varied from an actual ICU due to differences in time and stakes. As a result, transferable information from the simulation may be limited. In the simulation, for example, clinicians were assigned one patient at a time, removing the realism of a multi-patient workload. Also, the clinicians did not have access to other existing clinical information systems (e.g. EMR, monitoring and intervention medical device interfaces, and physical paper chart components) and an actual patient presenting physical symptoms. In addition, the data included artefacts inherent to medical device signal noise, and sharp peaks or dips in the data could not be verified as true values instead of false-positives or false-negatives. Again, scenarios were based on newborn patients who were post-cardiac surgery, limiting transferable information to other patient populations such as medical-surgical, trauma, or adult. However, we designed tasks using the software to be plausible to clinicians from both medical-surgical and cardiac specialties. Future work may include high-fidelity simulations with realistic patients; complementary technologies; a larger variety of reliable and validated scenarios; and, eventually, in-situ clinical simulations with appropriate metrics that replicate conditions for higher-level decision-making tasks [54, 55].

## Conclusions

Data integration and visualization software offers new ways of perceiving and interpreting data. Time series visual aids to represent continuous intensive care data were found to be satisfactory for single parameters but were less useful for multiparametric visualization and single point recall. Shading to highlight data overlaid on time series visualizations, as well as miniaturized time series (sparklines) with exaggerated extreme data values, were ignored. The multiparametric single indicator, which uses a visual aid to summarize the dynamic calculation of a 16-parameter algorithm, may support the dense use of data but should be tested further in the context of clinically relevant tasks. These findings highlight the importance and value of conducting usability testing to uncover potential ease of use and safety issues that can impact the acceptance of a data integration and visualization system. A recent review of 39 articles of physiologic data visualization found only one study on the usability of this type of software [56].

Our unique contributions to the study of interactive data integration systems is an understanding of how different clinical specialties interact with a commercial data integration and visualization technology. We also identified potential interface barriers to the use of such technology to each discipline-specific practice. The barriers include a difficulty with acquiring multiple parameter data from data-dense visualizations and perceiving out-of-target data. Another barrier is the limited clinical context of continuous data due to the separate medical recording systems (EMR). While this study was based specifically on the T3™ system, findings from this study may be applied generally to other data integration and visualization platforms. Specifically, the practical improvement suggestions may be applied to other platforms. For instance, features such as reducing redundant data streams and clinician cognitive demand in interacting with the visual displays can be effective in allowing clinicians to efficiently and easily abstract, trend, and interact with data thus resulting in improved clinician’s decision-making accuracy and efficiency.

Many opportunities exist to uncover other contributory factors beyond usability issues (e.g., perceived usefulness, implementation and change management strategy, and training) that can influence adoption of data integration technologies into clinical practice. Future research directions include the optimization of the software interface to improve data acquisition and interpretation; impact assessment of the optimized interfaces during realistic simulations; and, finally, naturalistic decision-making in the ICU setting. Design solutions, iteratively implemented and focused on the software system, are expected to mitigate use errors and promote the safe use of such novel software for intensive care. If tested in simulation, these solutions should be evaluated in a more realistic setting regarding environment and task load. Alternatively, solutions could be evaluated during use in the real ICU.

Intensive care clinicians must comprehensively integrate data from disparate technologies to closely monitor patients. The availability of multimodal continuous data may improve patient outcomes but risks being simply ignored, or worse, inadvertently introducing new problems such as cognitive overload that could lead to sub-optimal decision-making [3, 57]. Data integration software that enables real-time computation and visualization of continuous monitoring data are in rapid development [30, 47, 58, 59]. However, research has shown that poorly designed technologies lead to unintended issues, including cognitive overload, mental fatigue, and device recalls [38, 57, 60–66]. Grinspan et al. suggest that the ideal system should “allow clinicians to abstract, trend, and interact with copious amounts of data through an intuitive user interface” [10]. Moving forward, ICUs and vendors should consider how staff usability testing can assist selecting or customizing data integration software for improved acceptance of new technologies into high-risk, technologically-intense settings.

## Additional files

Additional file 1: List of usability tasks tested and representative questions posed to the participants. (DOCX 23 kb)

Additional file 2: Usability tasks tested with pass rates as percentage and fraction of total users. (DOCX 42 kb)

## Abbreviations

AH: Abstraction hierarchy
DR: Physician intensivist
EMR: Electronic medical record
ICU: Intensive care unit
MMM: Multimodal monitoring
RN: Registered nurse
RT: Respiratory therapist
UCD: User-centered design
UER: Use error rating
