# Supplementary material for: “Usability of data integration and visualization software for multidisciplinary pediatric intensive care: a human factors approach to assessing technology”
Source: BMC Med Inform Decis Mak. 2017 Aug 14;17:122. doi: 10.1186/s12911-017-0520-7 (PMC5557066; doi:10.1186/s12911-017-0520-7)
Supplement: Supplementary file 1 — List of usability tasks tested and representative questions posed to the participants. (DOCX 23 kb) [file 12911_2017_520_MOESM1_ESM.docx]

**Additional file 1**

List of usability tasks tested and representative questions posed to the participants. Checked box indicated a pass rate of less than 50%.

| **Task ID#** | **Optimistic** | | | **Conservative** | | | **Description of Tasks** | **Manuscript Description** | **Tasks/Questions Asked to Participant** |
| --- | --- | --- | --- | --- | --- | --- | --- | --- | --- |
|  | **DR** | **RN** | **RT** | **DR** | **RN** | **RT** |  |  |  |
| 1 |  |  |  |  |  |  | Locating patient file | 1. Locating patient file | Find patient file. |
| 2 |  |  |  |  |  |  | Recalling maximum or minimum values for a specific variable | 2. Identifying a value for a specific physiological variable | What was the lowest etCO_2_ value recorded during cardiac arrest? |
| 3 |  |  |  |  |  |  | Estimating duration of event | 3. Estimating duration of event by identifying two time points | How long after chest closure did the cardiac arrest happen? |
| 4 |  | ✓ | ✓ | ✓ | ✓ | ✓ | Time scale manipulation | 4. Manipulating time scale | How long has the patient been in the unit? |
| 5 |  |  |  |  |  |  | Comparing trends for two variables | 5. Comparing trends for two specific parameters | Did the blood pressure or oxygen saturation fall first? |
| 6 |  |  | ✓ |  |  |  | Comparing different patient states | 6. Comparing different patient physiological states | Provide values for HR and SpO_2_ pre- and post-surgery. How are these signals different from the current signals? |
| 7 |  | ✓ |  | ✓ | ✓ | ✓ | Recalling range of values for specific variables, surrounding a specific event | 7. Identifying values for two specific parameters at an event | What were the range of vitals for blood pressure and saturation during this event [cardiac arrest]? |
| 8 |  |  |  | ✓ | ✓ |  | Recalling change of values for several variables, prior to a specific event. Finding notes | 8. Identifying vital signs (group of parameters) prior to an event | Identify and report the vitals prior to the hypotension event post-surgery: hypotension event |
|  |  |  |  |  |  |  |  |  | Did these values change significantly after chest closure and prior to cardiac arrest? |
|  |  |  |  |  |  |  |  |  | Comment on the vitals you would use to indicate readiness for chest closure. |
| 9 |  |  |  | ✓ |  | ✓ | Selection of inactive variables. Time scale manipulation | 9. Viewing trend of three redundant, overlapping parameters | View and compare saturation data (SpO_2_, SpO_2_ r, SpO_2_ l) for entire length of stay. |
| 10 |  |  |  |  | ✓ |  | Viewing infusion rates over time | 10. Viewing infusion medication data | The dose of dopamine was increased over time. Please go back to the time period when this occurred. By how much and over what time period was dopamine increased? |
| 11 |  |  |  |  |  |  | Comparing infusions with vitals | 11. Comparing infusion medications with vital signs | What was the impact of the dopamine infusion on 1 or 2 vitals of concern? |
| 12 |  | ✓ |  |  | ✓ | ✓ | Viewing infusion data | 12. Detecting change in infusion medication rate over time | Around what time was epinephrine stopped? |
| 13 |  |  |  |  |  |  | Viewing ventilator data | 13. Viewing ventilator data | How did values of peak inspiratory airway pressure, mean airway pressure, and positive end expiratory pressure change during cardiac arrest? |
| 14 |  |  |  |  |  | ✓ | Viewing laboratory data | 14. Viewing laboratory data | What was the trend of the hematocrit, glucose, and PaCO_2_? |
| 15 | ✓ | ✓ | ✓ | ✓ | ✓ | ✓ | Visual representation of target ranges | 15. Viewing target ranges (semi-automated visual aid) | When were the targets set?  When did the values go out of range for these set targets? |
| 16 | ✓ |  | ✓ | ✓ | ✓ | ✓ | Sparklines | 16. Sparkline (automatic trend line for one variable) | Is there a faster way to visualize the trend for the past 30 minutes? How does the 30-minute automatic trend compare to the trends shown in the main graph? |
| 17 | ✓ |  | ✓ | ✓ | ✓ | ✓ | IDO_2_ indicator | 17. IDO_2_ indicator (automatic computation using 16 parameters) | Does this indicator signal approximate duration of patient instability? Is this a meaningful indication of patient instability? |
| 18 |  |  |  | ✓ |  |  | Finding notes | 18. Finding notes | A therapeutic intervention is required, please find this source of information and state the time of the intervention [inhaled nitric oxide]. |
|  |  |  |  |  |  |  |  |  | Approximately when did the physician attempt chest closure? |
|  |  |  |  |  |  |  |  |  | For ECMO initiation: A previous physician wrote a note indicating a therapeutic intervention, please find this source of information. |
| 19 |  |  |  | ✓ | ✓ |  | Modifying the patient record by adding note | 19. Modifying/adding note | The plan is to keep the patient hypothermic for 48 hours. Can you add a note indicating the start of hypothermia at 33 degrees Celsius? |
|  |  |  |  |  |  |  |  |  | After observing the effect of the intervention, please use T^3^ to communicate your thoughts on this event. |
| 20 |  |  |  |  |  |  | Setting targets | 20. Setting targets | Please set targets for oxygen saturation. |
|  |  |  |  |  |  |  |  |  | Assuming you are now coming off shift, please set an appropriate target range for heart rate to help with monitoring. |
| Total below 50% cut-off point | 3 | 4 | 5 | 9 | 9 | 8 |  |  |  |
