# Supplementary material for: “Usability of data integration and visualization software for multidisciplinary pediatric intensive care: a human factors approach to assessing technology”
Source: BMC Med Inform Decis Mak. 2017 Aug 14;17:122. doi: 10.1186/s12911-017-0520-7 (PMC5557066; doi:10.1186/s12911-017-0520-7)
Supplement: Supplementary file 2 — Usability tasks tested with pass rates as percentage and fraction of total users. (DOCX 42 kb) [file 12911_2017_520_MOESM2_ESM.docx]

Usability tasks tested with pass rates as percentage and fraction of total users.

| **General**  **Functions** | **Tasks Tested for Each Function** | **Pass rate by Task and by Clinician Type** | | | **Pass Rate by Task**  **(max n=22)** | **Usability Issue**  **(Y/N)** |
| --- | --- | --- | --- | --- | --- | --- |
|  |  | **Physicians**  **(max n=7)** | **Nurses**  **(max n=8)** | **Respiratory Therapists (max n=7)** |  |  |
| **Tracking:**  Orientation  (4 tasks) | 1. Locating patient file | 100% (7/7) | 100% (8/8) | 100% (7/7) | 100% (22/22) | N |
|  | 2. Identifying a value for a specific physiological variable | 80% (4/5) | 75% (3/4) | 67% (4/6) | 73% (11/15) | N |
|  | 3. Estimating duration of event by identifying two time points | 60% (3/5) | 100% (4/4) | 100% (5/5) | 86% (12/14) | N |
|  | 4. Manipulating time scale | 43% (3/7) | 0% (0/8) | 14% (1/7) | 18% (4/22) | Y |
|  | **Function Pass Rate by Clinician Type** | **71%** | **63%** | **68%** |  |  |
| **Trajectory:**  Relationships between Parameters  (10 tasks) | 5. Comparing trends for two specific parameters | 60% (3/5) | 57% (4/7) | 67% (4/6) | 61% (11/18) | N |
|  | 6. Comparing different patient physiological states | 67% (4/6) | 50% (4/8) | 50% (3/6) | 55% (11/20) | N |
|  | 7. Identifying values for two specific parameters at an event | 40% (2/5) | 43% (3/7) | 20% (1/5) | 35% (6/17) | Y |
|  | 8. Identifying vital signs (group of parameters) prior to an event | 17% (1/6) | 0% (0/8) | 50% (3/6) | 20% (4/20) | Y |
|  | 9. Viewing trend of three redundant, overlapping parameters | 29% (2/7) | 71% (5/7) | 17% (1/6) | 40% (8/20) | Y |
|  | 10. Viewing infusion medication data | 83% (5/6) | 29% (2/7) | 100% (5/5) | 67% (12/18) | N |
|  | 11. Comparing infusion medications with vital signs | 86% (6/7) | 83% (5/6) | 57% (4/7) | 75% (15/20) | N |
|  | 12. Detecting change in infusion medication rates over time | 57% (4/7) | 14% (1/7) | 0% (0/6) | 25% (5/20) | Y |
|  | 13. Viewing ventilator data | 100% (6/6) | 60% (3/5) | 80% (4/5) | 81% (13/16) | N |
|  | 14. Viewing laboratory data | 50% (3/6) | 75% (3/4) | 67% (4/6) | 63% (10/16) | N |
|  | **Function Pass Rate by Clinician Type** | **59%** | **45%** | **45%** |  |  |
| **Triggering**:  Automated  Integration  (3 tasks) | 15. Viewing target ranges (semi-automated visual aid) | 0% (0/5) | 20% (1/5) | 20% (1/5) | 13% (2/15) | Y |
|  | 16. Sparkline (automatic trend line for one variable) | 0% (0/5) | 0% (0/5) | 0% (0/4) | 0% (0/14) | Y |
|  | 17. IDO_2_ indicator (automatic computation using 16 parameters) | 20% (1/5) | 0% (0/8) | 0% (0/6) | 5% (1/19) | Y |
|  | **Function Use Error Rating, by Clinician Type** | **7%** | **6%** | **7%** |  |  |
| Other Functions  (3 tasks) | 18. Finding notes | 43% (3/7) | 88% (7/8) | 57% (4/7) | 64% (14/22) | N |
|  | 19. Modifying/adding note | 29% (2/7) | 29% (2/7) | 50% (3/6) | 35% (7/20) | Y |
|  | 20. Setting targets | 86% (6/7) | 86% (6/7) | 100% (6/6) | 90% (18/20) | N |
|  | **Function Pass Rate, by Clinician Type** | **52%** | **68%** | **68%** |  |  |
| **Number of Tasks with Usability Issues** |  | **9** | **9** | **8** |  |  |
| **All**  **functions** | **Global Function Pass Rate for All Functions and Clinicians by Clinician Type** | **54%** | **47%** | **49%** |  |  |

Groups highlighted in blue were below the 50% cut-off level.
